# Supplementary material for: Loss of the branched-chain amino acid transporter CD98hc alters the development of colonic macrophages in mice
Source: Commun Biol. 2020 Mar 18;3:130. doi: 10.1038/s42003-020-0842-3 (PMC7080761; doi:10.1038/s42003-020-0842-3)
Supplement: Supplementary file 1 — Supplementary information [file 42003_2020_842_MOESM1_ESM.pdf]

Supplementary Information

Supplementary Figures

Supplementary Figure 1. Characterization of bone marrow and colonic lamina propria monocytes and macrophages.

(**a**) Representative tSNE visualization of bone marrow cells displaying the indicated markers used for the identification of respective cell populations from one out of eight analyzed animals. (**b**) Staining control for figure 1d. (**c**) Representative tSNE visualization of colonic lamina propria (cLP) showing the distribution of indicated markers used for the identification of respective cell populations in the colonic lamina propria from one animal out of eight analyzed mice.

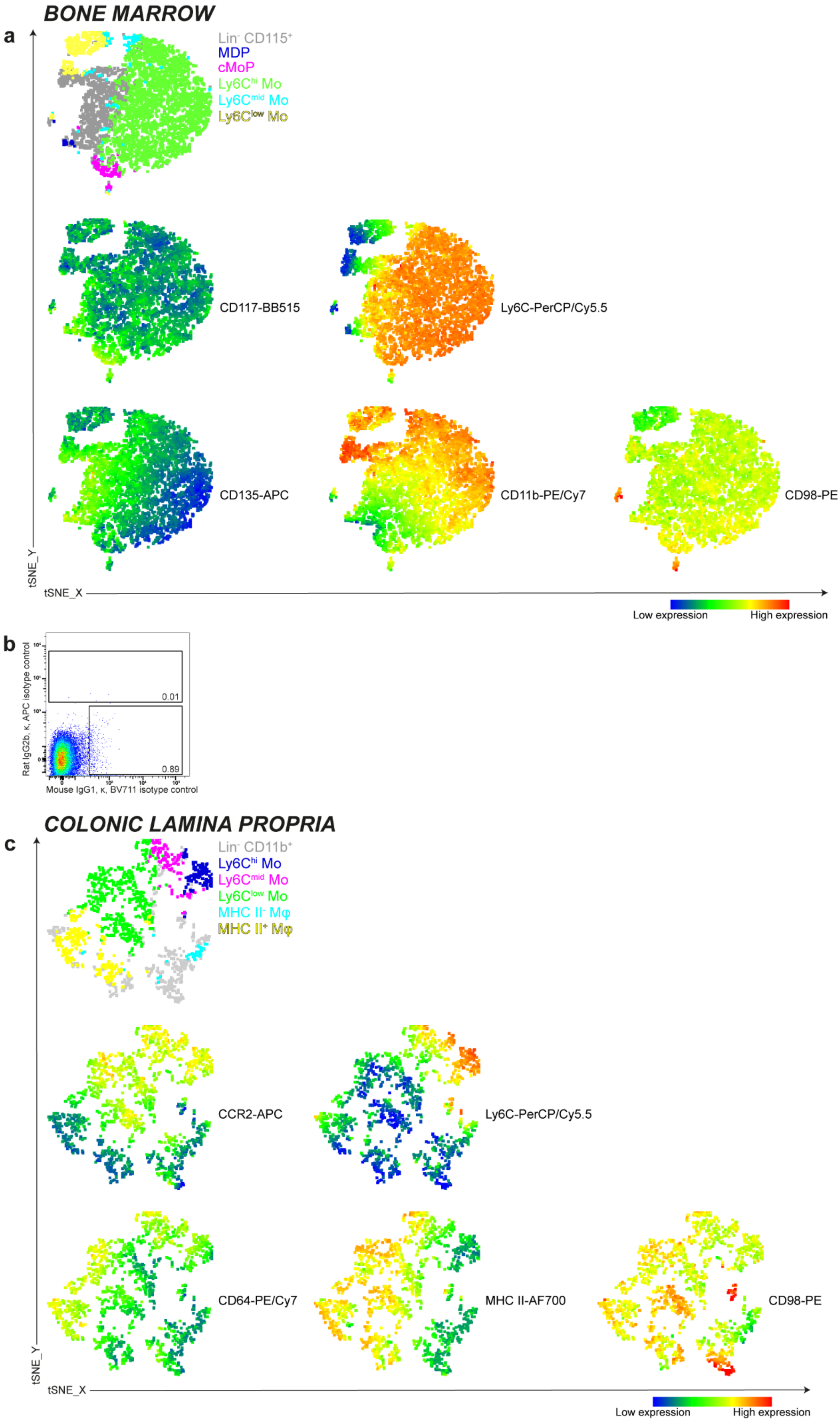

Supplement Figure 1

Supplementary Figure 2. CD98hc is expressed in the small and large intestine.

(**a**) The small intestine and the colon of naïve Cx3cr1-GFP mice were stained for CD98hc and counterstained with NucBlue. (**b** and **c**) Bone marrow derived macrophages (BMDM) were stimulated either with LPS + IFN-γ or with IL-4 + IL-13 and surface CD98 expression assessed by flow cytometry. Histogram and mean fluorescence intensity (MFI) are shown. (**d**) Histogram and (**e**) MFI of yolk sac macrophages from embryos (E8.5), $CD11c^-$ and $CD11c^+$ liver myeloid cells and Langerhans cells isolated from naïve adult mice and stained for CD98. Red histograms show FMO control, blue histograms cells stained for CD98. Number within plots indicated the percentage of $CD98^+$ macrophages, liver myeloid cells and Langerhans cells. (**f**) Gating strategy for the identification of the yolk sac macrophages, $CD11c^-$ and $CD11c^+$ liver myeloid cells and Langerhans cells. Statistical significance was calculated with a Kruskal-Wallis test followed by Dunn's correction; *$p<0.05$, **$p<0.01$. Experiments performed thrice for the CD98hc staining, thrice for the CD98hc expression in fetus and Langerhans cells, and seven times for liver myeloid cells.

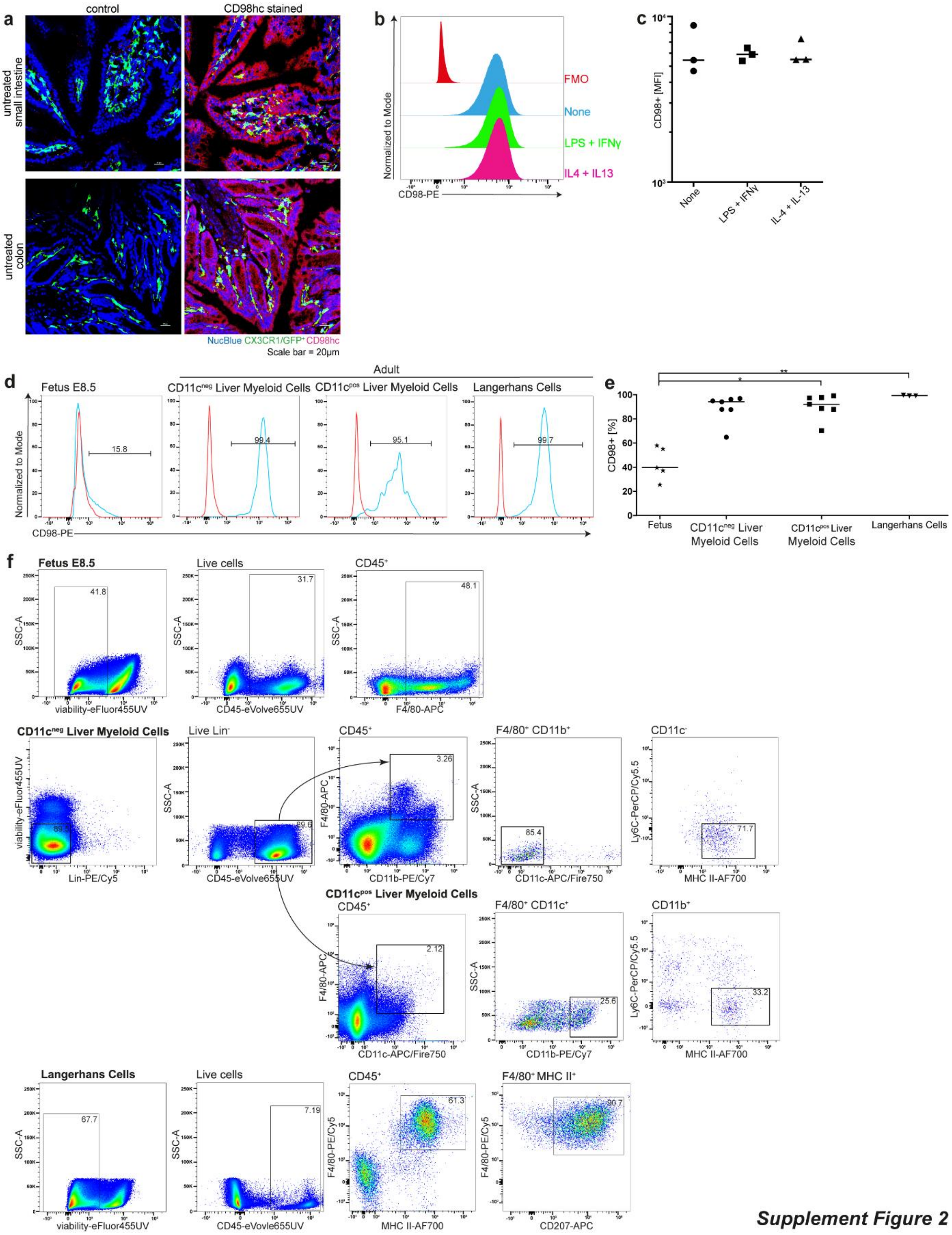

Supplement Figure 2

Supplementary Figure 3. Tamoxifen induced Cre recombinase deletes CD98hc in monocytes and macrophages but not in T cells in CD98hc$^{\Delta CX3CR1}$ animals.

(**a**) CD98hc$^{flox/flox}$ mouse construct that were breed with Cx3cr1^{CreER} mice. (**b**) Genotyping of, CD98hc$^{flox/flox}$, CD98hc$^{\Delta CX3CR1}$ and wildtype (WT) animals. (**c**) Percentage of CD98 expression by B cells, CD4^{+} and CD8^{+} T cells, dendritic cells (DC), macrophages, neutrophils isolated from the colonic lamina propria of corn oil- or tamoxifen-treated C57BL/6 WT or CD98hc$^{\Delta CX3CR1}$ animals. (**d**) Gating strategy for the identification of B cells, CD4^{+} and CD8^{+} T cells, dendritic cells (DC), macrophages, neutrophils isolated from the colonic lamina propria. (**e**) Percentage of integrin β1/CD29^{+} monocytes and macrophages in indicated animals treated with corn oil or tamoxifen. Data was analyzed by two-way ANOVA followed by Sidak's correction; *$p<0.05$, ****$p<0.0001$. Experiments performed twice.

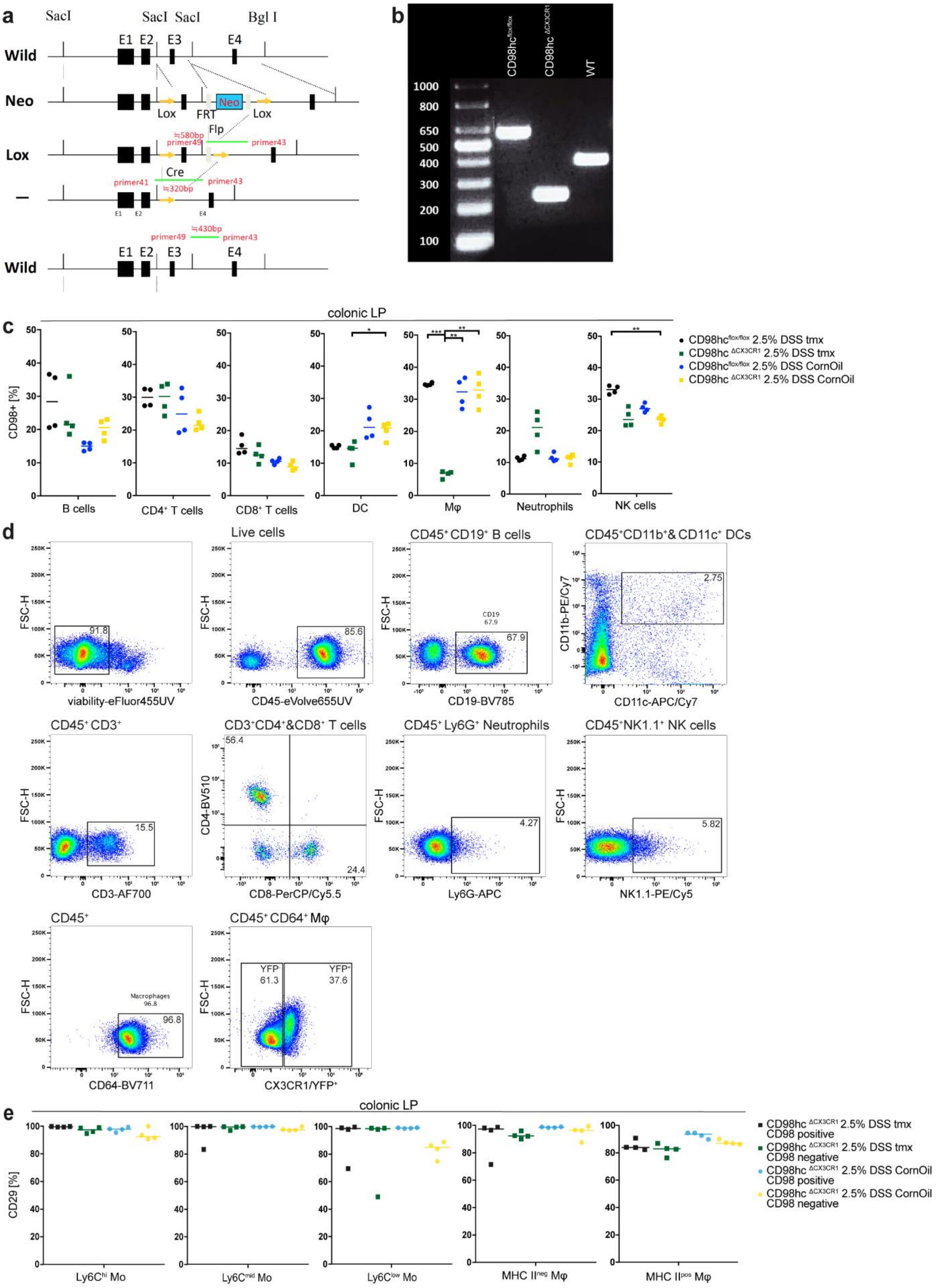

Supplement Figure 3

Supplementary Figure 4. CD98hc deletion in cardiac macrophages but not in microglia. (**a**) Gating strategy for the identification of microglia and cardiac macrophages obtained after exclusion of dead cells. Red histograms show FMO control, blue histograms present cells stained for CD98hc. Circulating cell of hematopoietic origin were excluded after i.v. injection of an anti-CD45 SuperBright antibody into the animals at least 5 min before isolation. (**b**) Absolute cell numbers of microglia and cardiac macrophages and percentage of CD98hc expressing macroglia and cardiac macrophages after intraperitoneal corn oil or tamoxifen injection in CD98hc$^{\Delta CX3CR1}$ mice (n=3) and analyzed by two-way ANOVA followed by Sidak's correction; *p<0.05. Experiments performed once with three biological replicates.

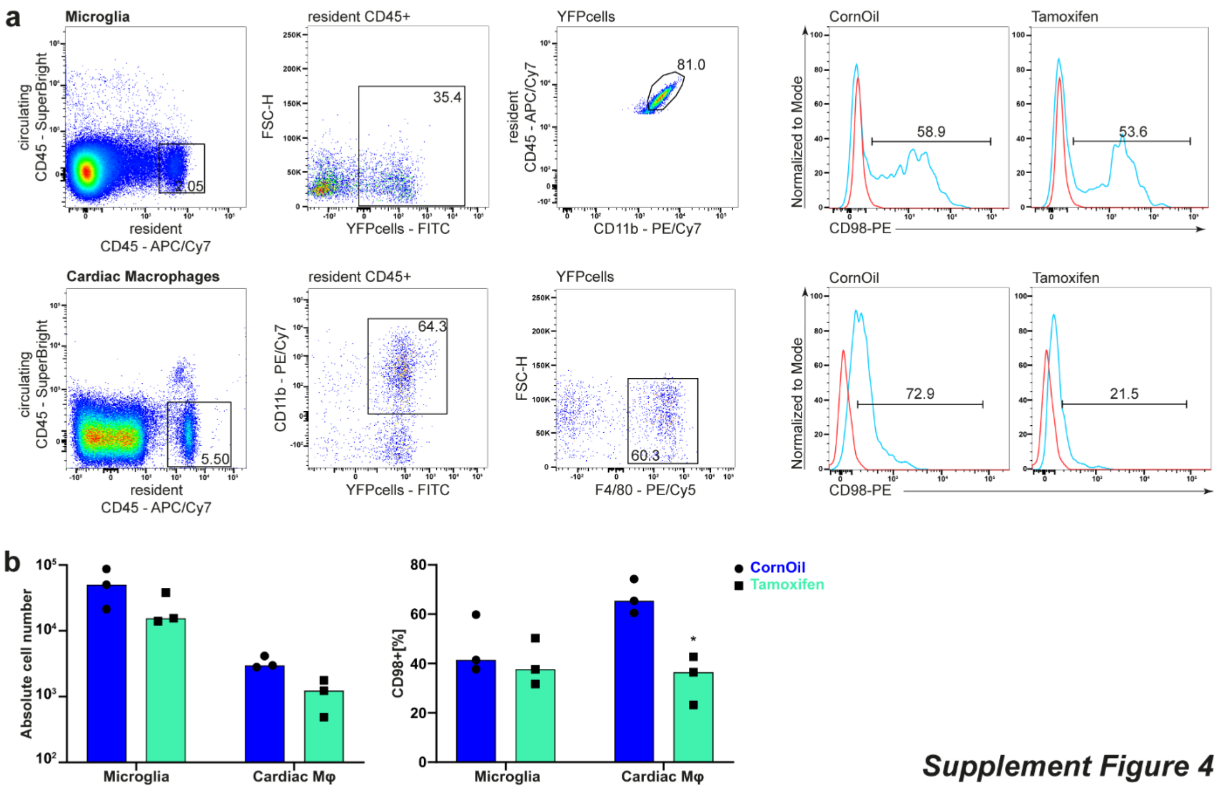

Supplement Figure 4

Supplementary Figure 5. Animals fed with regular chow or L-leucin/L-isoleucine enriched chow before and after the deletion of CD98hc during DSS-induced colitis.

Colitis was induced by adding 2.5% dextran sodium sulfate (DSS) to CD98hc$^{flox/flox}$ and CD98hc$^{\Delta CX3CR1}$ mice which were treated either with corn oil or tamoxifen two days before DSS administration and fed with either regular chow or regular chow enriched with 5% L-leucine and 5% L-isoleucine. (**a**) Disease activity index, colon length, and histological scores of indicated groups. The data were analyzed by two-way ANOVA followed by Sidak's correction; ****p<0.0001. Histological scores were assessed in a blinded fashion by two independent investigators. The mean histological score was determined for each animal after H&E staining of colonic tissues, presented as individual dot and analyzed with a Mann-Whitney U test; *p<0.05. The colon length was determined at day 7 after start of DSS administration, colon length is shown for each individual animal, the mean indicated and analyzed with a Mann-Whitney U test; *p<0.05. (**b**) A representative image of the colon from each group is shown. (**c**) H&E staining of colonic tissues. (**d**) A representative image of the colonic CX3CR1/YFP$^+$ cells counterstained with NucBlue. (**e**) Quantification of the colonic CX3CR1/YFP$^+$ cells per high power field (HPF) per mouse. The Experiment was performed once.

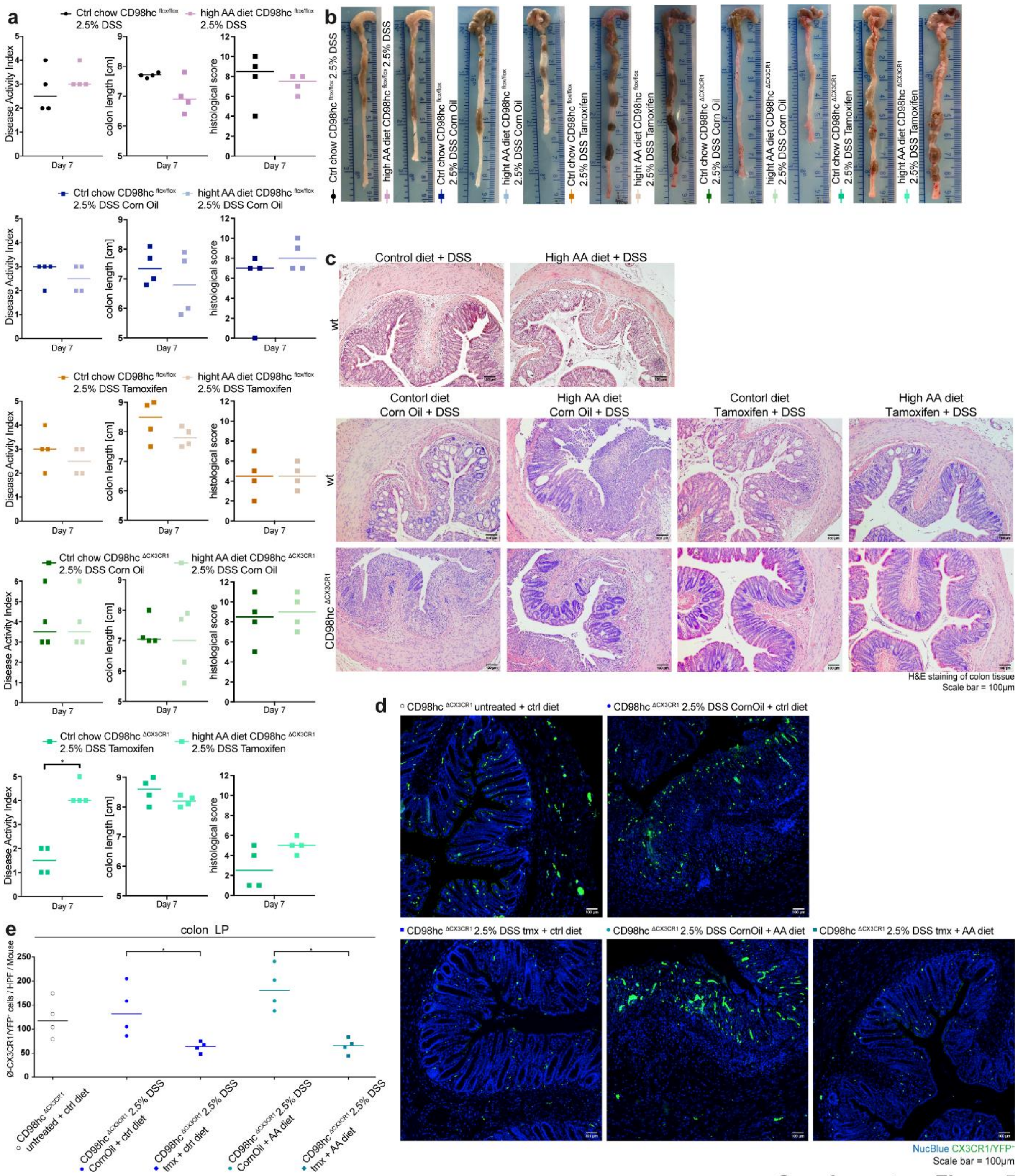

Supplementary Figure 5

Supplementary Figure 6. mTORC1 pathway is not influenced by CD98hc deletion.

(**a**) Colitis was induced by adding 2.5% dextran sodium sulfate (DSS) to CD98hc$^{flox/flox}$ and CD98hc$^{\Delta CX3CR1}$ mice which were treated either with corn oil or tamoxifen two days before DSS administration. Histogram of the phosphorylation of p70S6K. Numbers indicate the percentage of positive cells. (**b**) Percentage of phosphorylated p70S6K and S6 in bone marrow derived macrophages (BMDM), which were cultured in RPMI 1640 medium containing essential amino acids (EAA), EAA and either LPS + IFNγ or IL-4 + IL-13 stimulation, non-essential amino acid (NEAA) medium, and NEAA supplemented with L-leucine (0.05 g/L). (**c**) Changes in normalized p70S6K and S6 phosphorylation in BMDM. (**d**) qRT-PCR of indicates cytokines, *iNos*, *Mcp1* and *Kc* of BMDM cultured in EAA, EAA and LPS + IFNγ stimulated, NEAA, and NEAA supplemented with L-leucine (0.05 g/L). Experiment performed with three biological replicates or in duplicates, respectively.

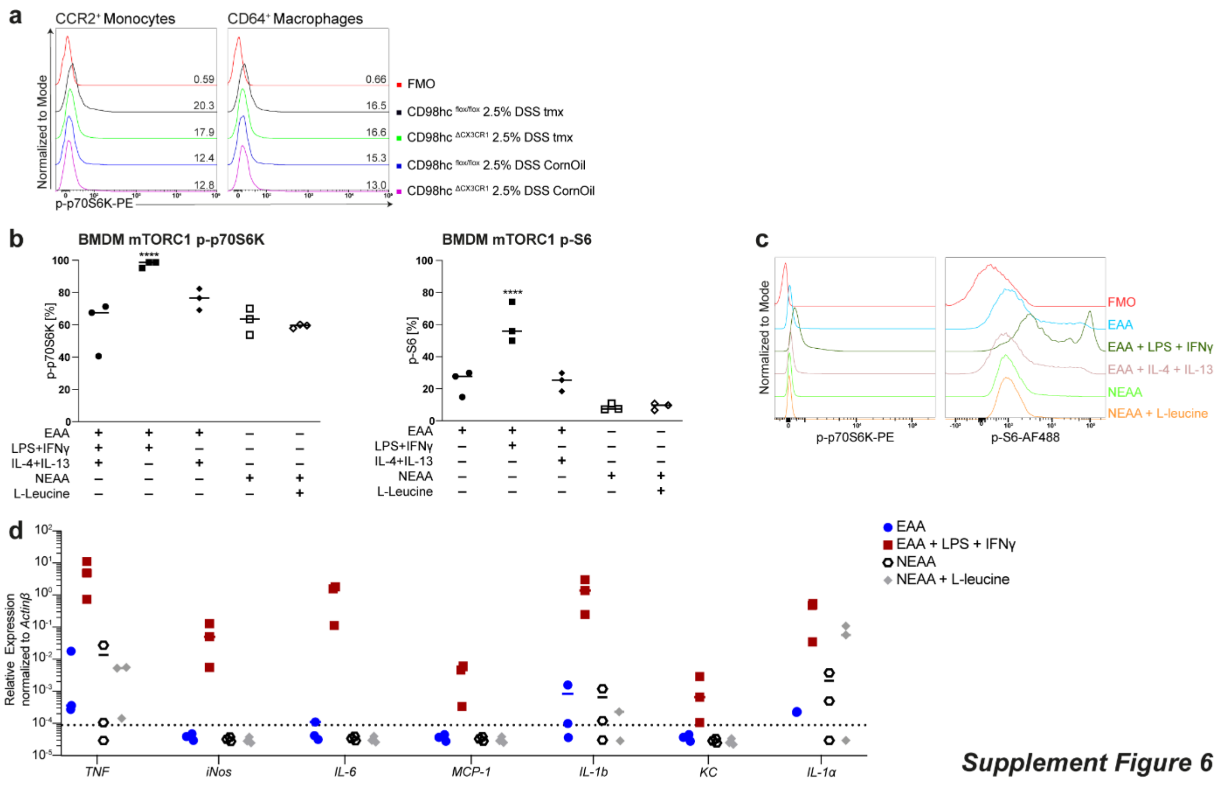

Supplement Figure 6

Supplementary Figure 7. Verification of relevant patterns observed in the scRNA-seq data.

(**a**) tSNE plots of control (corn oil-treated CD98hc$^{\Delta CX3CR1}$ mice) and CD98hc cKO (tamoxifen-treated CD98hc$^{\Delta CX3CR1}$ mice) and control and CD98hc cKO tSNE plots, where the individual samples per condition have been annotated. (**b**) Heatmap of cluster specific genes by cells from corn oil (control) and tamoxifen (cKO) treated animals. The clusters are shown in different colors. Heatmaps are showing the centered and scaled average expression (normalized log-counts values) across cells of each cluster in each condition. The experiment was performed once (scRNA-seq) with four biological replicates per group.

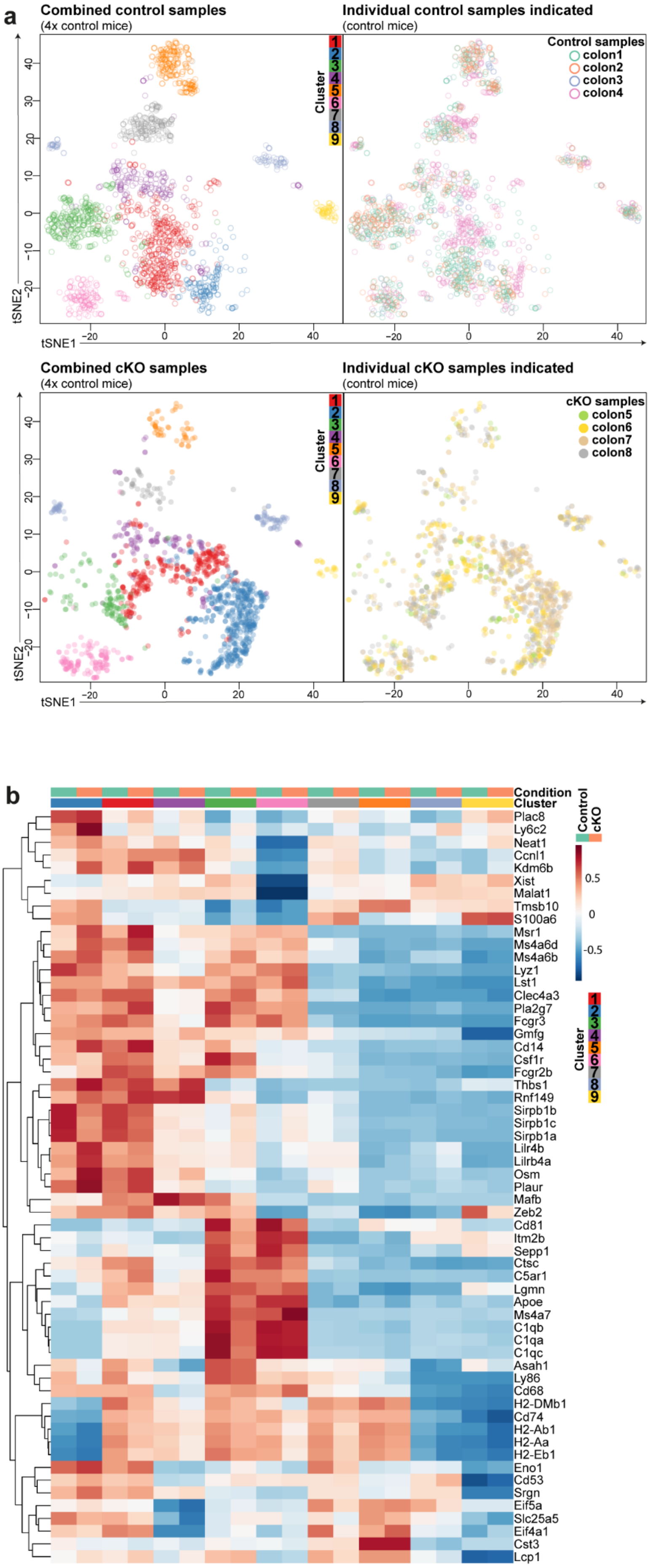

114 ***Supplement Figure 7***

Supplementary Figure 8. tSNE visualization of the expression of arbitrarily chosen markers.

The experiment was performed once (scRNA-seq) with four biological replicates per group.

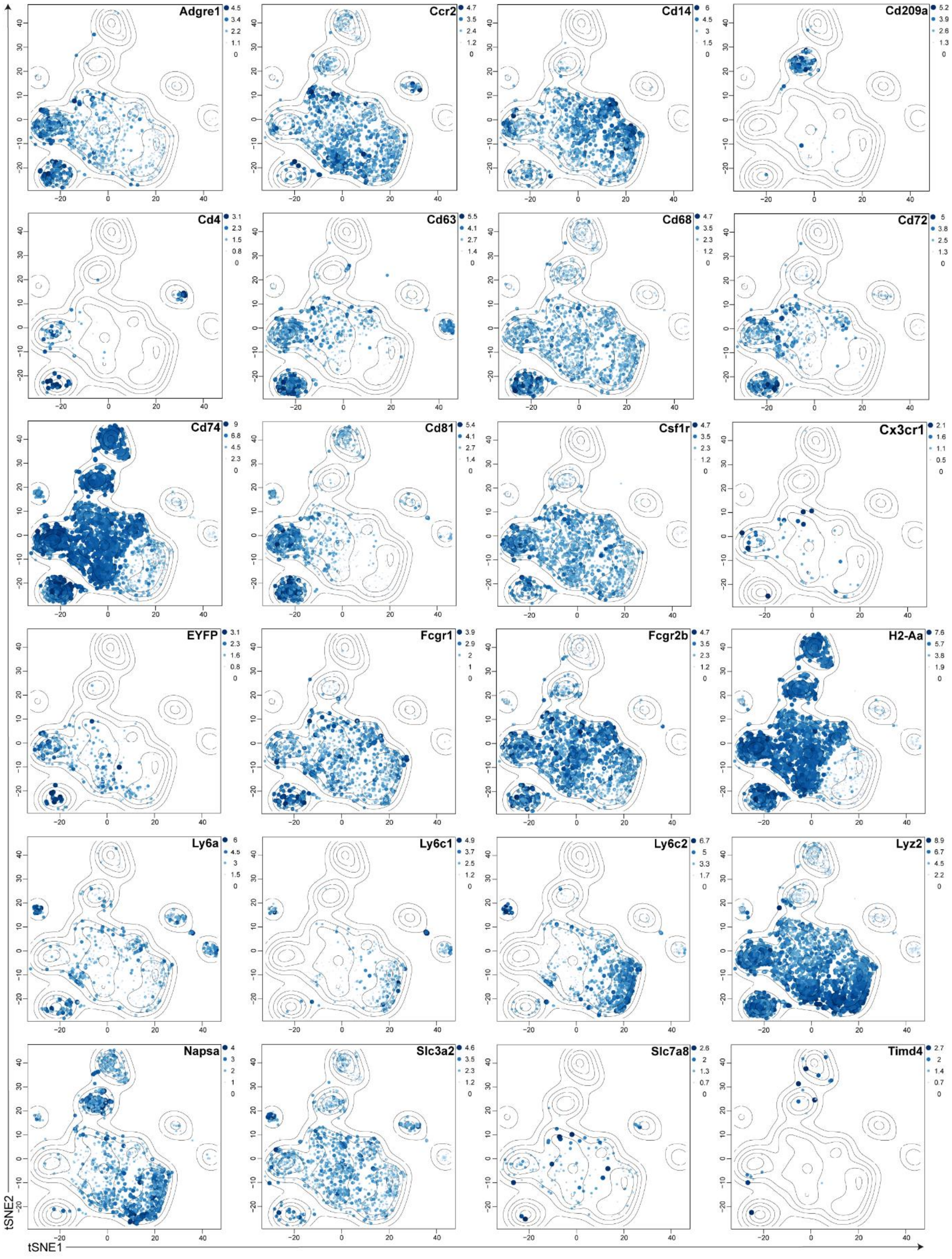

Supplement Figure 8

Supplementary Figure 9. Chosen marker expression by flow cytometry.

(**a**) Percentage of $CD14^+$, $CD81^+$, and $CD72^+$ colonic lamina propria monocytes and macrophages as determined by flow cytometry. Each dot represents an individual animal and the median value is shown. Data was analyzed by two-way ANOVA followed by Sidak's correction; ***p<0.001, ****p<0.0001. (**b**) Histogram plot of monocytes and macrophages after CD14, CD81, and CD72 staining. Red line represents the FMO control and blue line shows stained cells. Numbers in histograms indicate the percentage of $CD14^+$, $CD81^+$, and $CD72^+$ monocytes and macrophages. (**c**) Relative frequency of control and cKO cells within the different clusters defined based on the scRNA-seq experiment. Experiments performed once with three to four biological replicates per group.

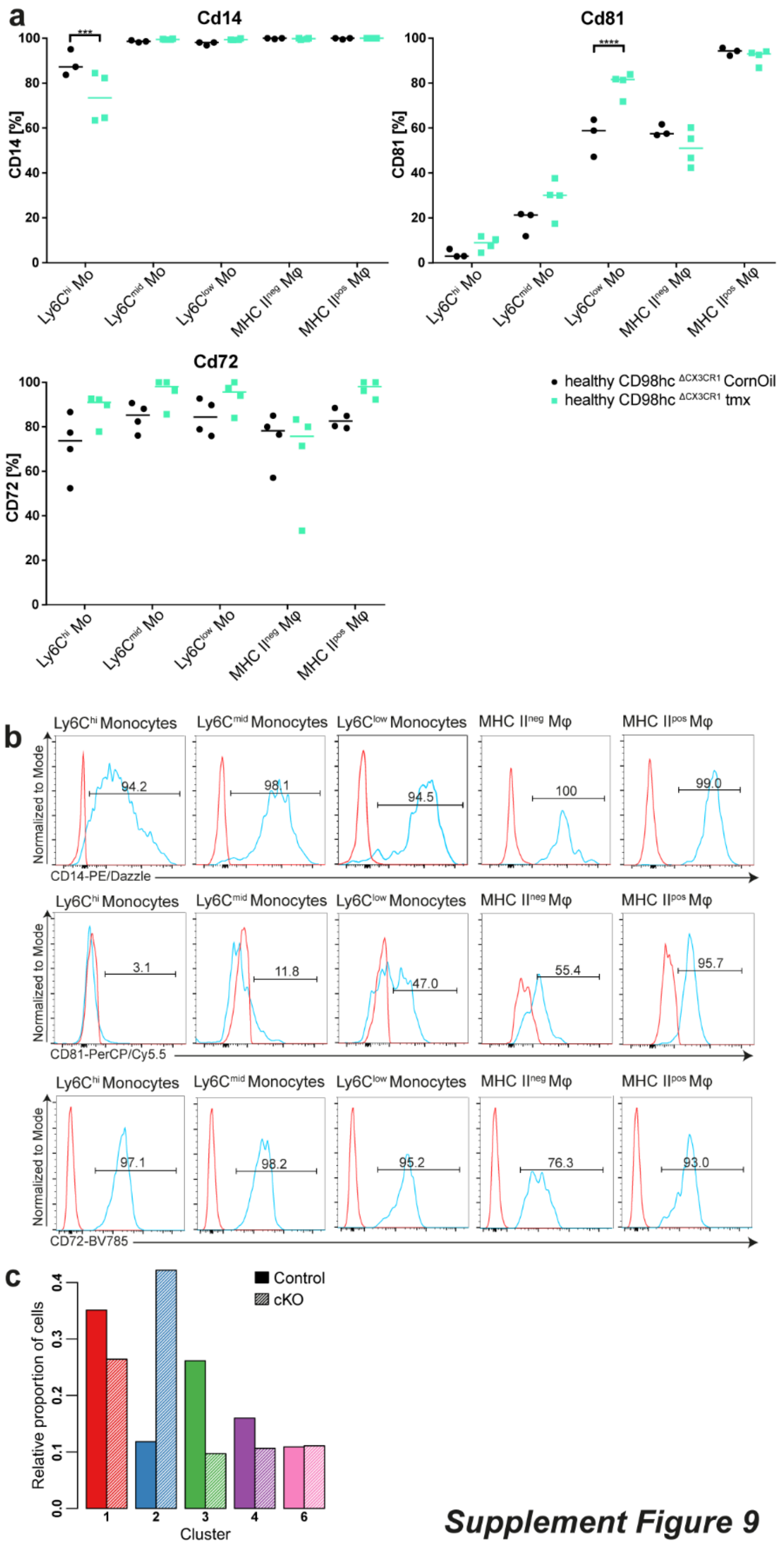

Supplement Figure 9

Supplementary Figure 10. Deletion of CD98hc leads to increased apoptosis and reduced cell number in colonic macrophages.

(**a**) Percentage of apoptotic cells determined by flow cytometry after Annexin V/viability staining by indicated monocytes and macrophages isolated from corn oil- or tamoxifen treated CD98hc$^{flox/flox}$ or CD98hc$^{\Delta CX3CR1}$ animals. (**b**) Annexin V/viability staining of indicated experimental groups are presented by dot plot graphs. Data is shown as the mean and analyzed by two-way ANOVA followed by Sidak's correction; ***$p<0.001$, ****$p<0.0001$. The experiment was performed once.

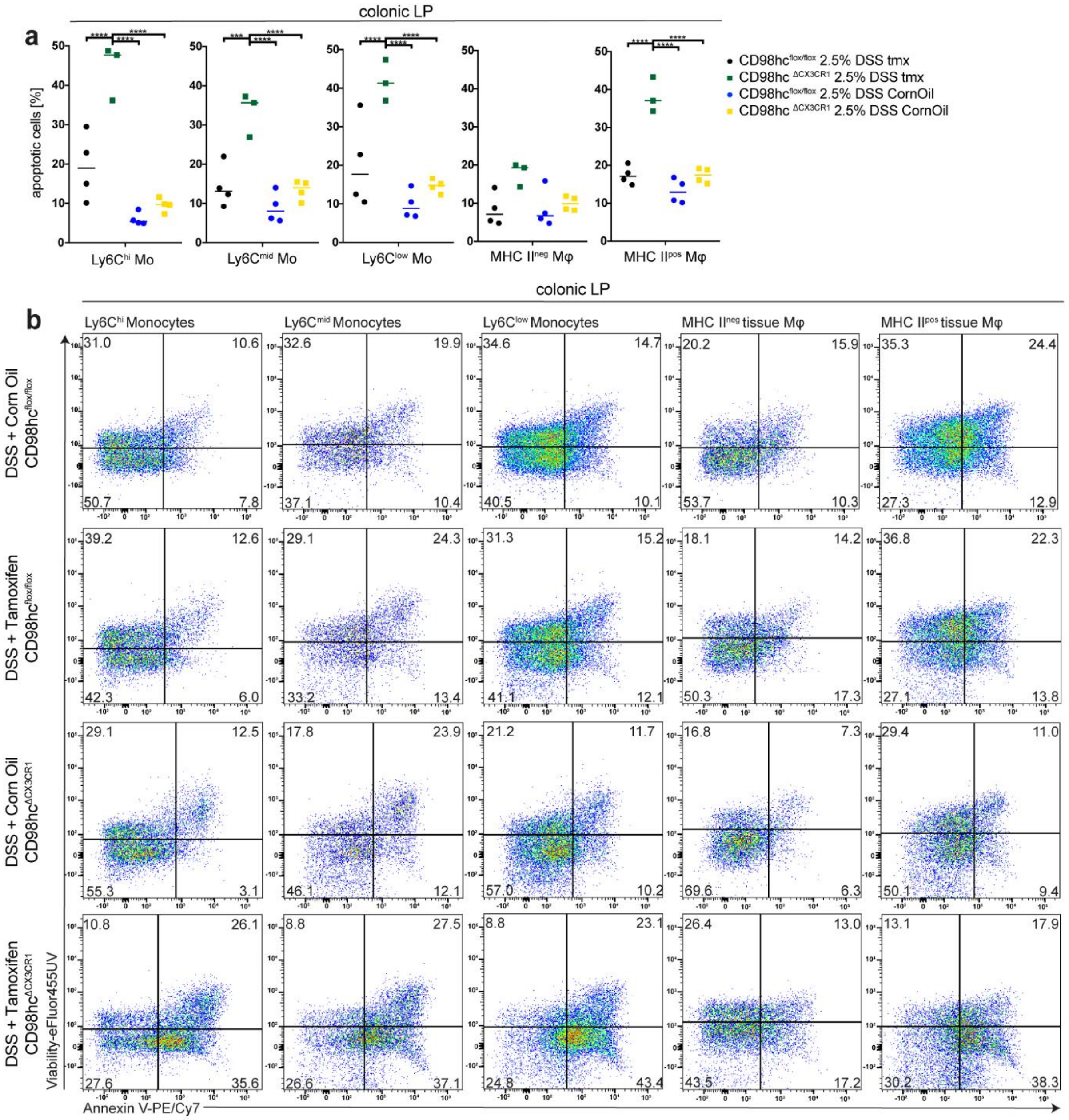

Supplement Figure 10

Supplementary Figure 11. ***In vitro*** **deletion of CD98hc results in BMDM cell death.**

Non-stimulated, LPS + IFNγ, or IL-4 + IL-13 stimulated bone marrow-derived macrophages (BMDM) were cultured in RPMI 1640 medium containing: none (control), 100 µg dimethyl sulfoxide (DMSO control), 2.5 µg, 5 µg, and 10 µg tamoxifen dissolved in DMSO. Annexin V/viability and CD98 staining were performed and analyzed by flow cytometry to assess dead or apoptotic cells along with CD98 expression. The experiment was performed thrice.

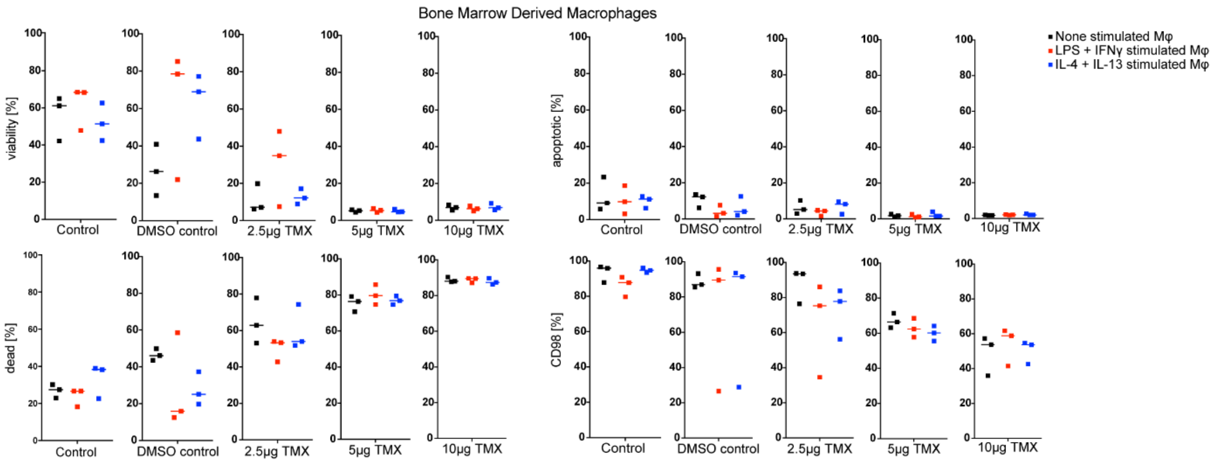

Supplement Figure 11

Supplementary Figure 12.

Schematic representation of the ‘monocyte waterfall’-development to gut macrophages in C57BL/6 WT and CD98hc deficient mice.

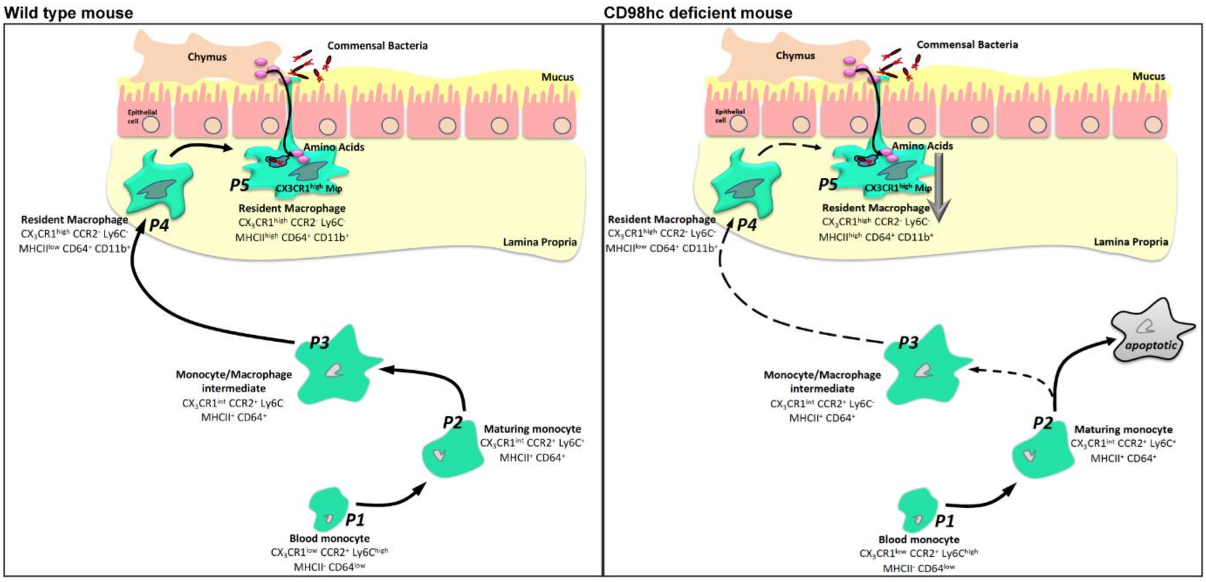

Supplement Figure 12

Supplementary Tables

Supplementary Table 1.

Depicted 10× genomics web summaries.

Treatment	CD98hc$^{\Delta CX3CR1}$ Colon #	Estimated Number of Cells	Mean Reads per Cell	Median Genes per Cell
Corn Oil	1	696	63.153	1.657
	2	495	74.116	1.794
	3	94	320.447	1.630
	4	536	53.893	1.666
Tamoxifen	5	95	455.324	1.476
	6	387	103.541	1.378
	7	506	65.280	1.680
	8	435	97.441	1.614

Supplementary Table 2.

Annotated cell types per cluster.

Source Name	Cell Type
Cluster 1	Monocytes, Macrophages
Cluster 2	Monocytes
Cluster 3	Macrophages
Cluster 4	Monocytes, Macrophages, Endothelial cells, Epithelial cells
Cluster 5	Dendritic cells
Cluster 6	Macrophages
Cluster 7	Monocytes, Macrophages, Dendritic cells
Cluster 8	B cells, Innate lymphoid cells (ILC), T cells
Cluster 9	Fibroblast, Stromal cells

Supplementary Table 3.

Characteristics of healthy patients and patients with Crohn’s disease (CD) or ulcerative colitis (UC), who provided biopsies to the Swiss IBD cohort used for *CD98hc/SLC3A2* and *CD98lc/SLC7A5* expression analysis.

**Baseline Group characteristics**					
	Healthy	***Crohn’ disease***		***Ulcerative colitis***	
	n=7	**Quiescent (n=20)**	**Active (n=11)**	**Quiescent (n=20)**	**Active (n=11)**
Gender, male/female, n (%)	71/29%<br>5 m, 2 f	60/40 %<br>12 m, 8 f	36/64%<br>4 m, 7 f	35/65%<br>12 m, 8 f	54/45%<br>6 m, 5 f
Median age (range), yr	42 (18-52)	56 (32-81)	39 (31-71)	58 (31-81)	53 (24-68)
Mean BMI (SD), kg/m^2	23,6 (2,57)	24.15 (3)	25.55 (4.9)	24.70 (2.99)	23.89 (4.08)
Median age at diagnosis (range), yr	–	33.5 (18-60)	21 (14-34)	28.5 (14-65)	30 (16-60)
Median disease duration (range), yr	–	21.5 (6-45)	19 (7-37)	26 (8-44)	16 (7-38)
	Healthy	**CD extent, n (%)**			
Ileum isolated	–	5 (25%)	2 (18%)	–	–
Colon isolated	1 (14%)	2 (10%)	3 (27%)	–	–
Ileocolonic	1 (14%)	2 (10%)	5 (46%)	–	–
unknown	–	11 (55%)	1 (9%)	–	–
Colon ascending	2 (29%)	–	–	–	–
Sigmoid	1 (14%)	–	–	–	–
random	2 (29%)	–	–	–	–
	Healthy	**UC extent, n (%)**			
Proctitis	–	–	–	1 (5%)	1 (10%)
Left-sided colitis	–	–	–	3 (20%)	5 (45%)
Pancolitis	–	–	–	5 (25%)	5 (45%)
Unknown	–	–	–		
	Healthy	**Current medical treatment, n (%)**			
No treatment	–	5 (25%)	–	5 (25%)	1 (9%)
5-ASA	–	6 (30%)	–	13 (65%)	9 (81%)
Steroids	–	–	6 (54%)	–	4 (36%)
Immunosuppressants	–	11 (55%)	1 (9%)	4 (20%)	1 (9%)
Anti-TNF	–	–	1 (9%)	–	3 (27%)
**Smoking status**					
Non-smoker, n (%)	1 (14%)	12 (60%)	6 (54%)	15 (75%)	9 (81%)
Active smoker, n (%)	3 (43%)	6 (30%)	5 (46%)	1 (5%)	1 (9.5%)
unknown	3 (43%)	2 (10%)	0	4 (20%)	1(9.5%)

Supplementary Table 4.

Characteristics of healthy patients and patients with IBD of the Basler IBD cohort, biopsy donors form healthy, inflamed, and non-inflamed regions for immunofluorescence staining.

	Healthy					***Ulcerative colitis***			***Crohn's disease***		
**Sample #**	*5266*	*5268*	*5269*	*5270*	*5272*	*504*	*535*	*619*	*558*	*568*	*620*
**Gender**	male	female	female	male	male	female	female	unknown	female	male	female
**Age**	61	30	61	42	58	50	71	unknown	72	68	69
**BMI**	20,1	unknown	23,5	31,7	30,6	25,6	27,3	unknown	19	32	21,6
**Age at diagnosis**	–					34	56	unknown	23	52	56
**Smoking status**	unknown	unknown	Yes	former	former	unknown	unknown	unknown	non-smoker	active	active
**Localisation inflamed**	–					sigmoid/ rectum	rectum/ sigmoid	sigmoid/ rectum	unknown	sigmoid	terminal Ileum
**Localisation non-inflamed**	–					transversal colon	colon	ascending/ transversal colon	rectum	colon descending	colon ascending
**Medical treatment at time of study**	–					none	none	none at time of study, but received Salofalk 10 days before	Quantalan, Immodium	Spiricort, Aldactone, Orfiril	unknown
**Clinical disease activity index**	–					5	6	unknown	unknown	74	70

Note: age calculated as of 2019 (healthy) and 2018 (IBD patient), not the year of sample

Supplementary Table 5.

Primers used for qPCR in this study.

**Primer (mouse)**	**Sequence (5ʹ-3ʹ)**	**Tm (°C)**	**Product size (bp)**
Actβ-fwd	TTC TTT GCA GCT CCT TCG TT	56,4	149
Actβ-rev	ATG GAG GGG AAT ACA GCC C	59,5	
Il1β-fwd	TGT GAA ATG CCA CCT TTT GA	54,3	94
Il1β-rev	GGT CAA AGG TTT GGA AGC AG	58,4	
Il6-fwd	TCG GAG GCT TAA TTA CAC ATG TTC T	62,5	94
Il6-rev	GCA TCA TCG TTG TTC ATA CAA TCA	60,3	
Mcp-1-fwd	AGG TCC CTG TCA TGC TTC TG	60,5	249
Mcp-1-rev	TCT GGA CCC ATT CCT TCT TG	58,4	
Tnf-fwd	CCA CCA CGC TCT TCT GTC TAC	63,2	103
Tnf-rev	AGG GTC TGG GCC ATA GAA CT	60,5	
Il-1α-fwd	CGC TTG AGT CGG CAA AGA AAT	59,5	271
Il-1α-rev	CTT CCC GTT GCT TGA CGT TG	60,5	
iNos-fwd	GTT CTC AGC CCA ACA ATA CAA GA	60,9	127
iNos-rev	GTG GAC GGG TCG ATG TCA C	61,6	
Kc-fwd	CTG GGA TTC ACC TCA AGA ACA TC	62,9	117
Kc-rev	CAG GGT CAA GGC AAG CCT C	61,6	
**Primer (human)**	**Sequence (5ʹ-3ʹ)**	**Tm (°C)**	**Product size (bp)**
GAPDH-fwd	TCG ACA GTC AGC CGC ATC TTC TTT	65,2	104
GAPDH-rev	GCC CAA TAC GAC CAA ATC CGT TGA	65,2	
SLC3A2-fwd	GAC CCC TGT TTT CAG CTA CG	60,5	108
SLC3A2-rev	TCA GGG AAG CTG GAC TCA TC	60,5	
SLC7A5-fwd	TCC TGG ATC ATC CCC GTC TT	60,5	88
SLC7A5-rev	CCA CGA AGA AGA GCC TGG AG	62,5	
