# Supplementary material for: Loss of the branched-chain amino acid transporter CD98hc alters the development of colonic macrophages in mice
Source: Commun Biol. 2020 Mar 18;3:130. doi: 10.1038/s42003-020-0842-3 (PMC7080761; doi:10.1038/s42003-020-0842-3)
Supplement: Supplementary file 2 — Description of Additional Supplementary Files [file 42003_2020_842_MOESM2_ESM.pdf]

Description of additional supplementary items

Supplementary Data 1 (10× genomics web summaries)

Diagnostic plots for the 10× genomics sequencing of WT and cKO cells.

Supplementary Data 2

Reference datasets retrieved from ImmGen used to identify the cell types per cluster.

Supplementary Data 3 (Plotly Visualization)

3D-visualization of the scRNA-seq principle component analysis.

Supplementary Data 4

Differentially expressed genes in CD98hc cKO cells over control cells for each cluster of the scRNA-seq data.

Supplementary Data 5

Source data underlying the graphs in figures and supplementary material as a n excel file.
