# Supplementary material for: Loss of the branched-chain amino acid transporter CD98hc alters the development of colonic macrophages in mice
Source: Commun Biol. 2020 Mar 18;3:130. doi: 10.1038/s42003-020-0842-3 (PMC7080761; doi:10.1038/s42003-020-0842-3)
Supplement: Supplementary file 8 — Reporting Summary [file 42003_2020_842_MOESM8_ESM.pdf]

nature research

Corresponding author(s): Dr. Jan Hendrik Niess

Last updated by author(s): Dec 26, 2019

Reporting Summary

Nature Research wishes to improve the reproducibility of the work that we publish. This form provides structure for consistency and transparency in reporting. For further information on Nature Research policies, see Authors & Referees and the Editorial Policy Checklist.

Statistics

For all statistical analyses, confirm that the following items are present in the figure legend, table legend, main text, or Methods section.

n/a	Confirmed	
☐	☒	The exact sample size (*n*) for each experimental group/condition, given as a discrete number and unit of measurement
☐	☒	A statement on whether measurements were taken from distinct samples or whether the same sample was measured repeatedly
☐	☒	The statistical test(s) used AND whether they are one- or two-sided<br>*Only common tests should be described solely by name; describe more complex techniques in the Methods section.*
☐	☒	A description of all covariates tested
☐	☒	A description of any assumptions or corrections, such as tests of normality and adjustment for multiple comparisons
☐	☒	A full description of the statistical parameters including central tendency (e.g. means) or other basic estimates (e.g. regression coefficient) AND variation (e.g. standard deviation) or associated estimates of uncertainty (e.g. confidence intervals)
☐	☒	For null hypothesis testing, the test statistic (e.g. *F*, *t*, *r*) with confidence intervals, effect sizes, degrees of freedom and *P* value noted<br>*Give P values as exact values whenever suitable.*
☒	☐	For Bayesian analysis, information on the choice of priors and Markov chain Monte Carlo settings
☒	☐	For hierarchical and complex designs, identification of the appropriate level for tests and full reporting of outcomes
☒	☐	Estimates of effect sizes (e.g. Cohen's *d*, Pearson's *r*), indicating how they were calculated

Our web collection on statistics for biologists contains articles on many of the points above.

Software and code

Policy information about availability of computer code

Data collection	FACSDiva, FlowJo
Data analysis	GraphPad PRISM, Excel, ImageJ, FlowJo, FACSDiva, Adobe Illsutrator, NIS-Elements C, BioStation IM, QuantStudio™ Real-Time PCR

For manuscripts utilizing custom algorithms or software that are central to the research but not yet described in published literature, software must be made available to editors/reviewers. We strongly encourage code deposition in a community repository (e.g. GitHub). See the Nature Research guidelines for submitting code & software for further information.

Data

Policy information about availability of data

All manuscripts must include a data availability statement. This statement should provide the following information, where applicable:

- Accession codes, unique identifiers, or web links for publicly available datasets
- A list of figures that have associated raw data
- A description of any restrictions on data availability

The scRNA-seq data have been uploaded at GEO (https://www.ncbi.nlm.nih.gov/geo/query/acc.cgi?acc=GSE126574)

Field-specific reporting

Please select the one below that is the best fit for your research. If you are not sure, read the appropriate sections before making your selection.

☒ Life sciences ☐ Behavioural & social sciences ☐ Ecological, evolutionary & environmental sciences

For a reference copy of the document with all sections, see nature.com/documents/nr-reporting-summary-flat.pdf

Life sciences study design

All studies must disclose on these points even when the disclosure is negative.

Sample size	Number of animals are given; specific sample size calculations before the experiment were not carried out.
Data exclusions	All obtained data were included
Replication	Experiments were replicated multiple times
Randomization	Randomization for animal selection was not persued
Blinding	Scoring of animals and asessemnt of histological scores was carried out in a blinded fashion

Reporting for specific materials, systems and methods

We require information from authors about some types of materials, experimental systems and methods used in many studies. Here, indicate whether each material, system or method listed is relevant to your study. If you are not sure if a list item applies to your research, read the appropriate section before selecting a response.

Materials & experimental systems

n/a	Involved in the study	
☐	☒	Antibodies
☒	☐	Eukaryotic cell lines
☒	☐	Palaeontology
☐	☒	Animals and other organisms
☐	☒	Human research participants
☒	☐	Clinical data

Methods

n/a	Involved in the study	
☒	☐	ChIP-seq
☐	☒	Flow cytometry
☒	☐	MRI-based neuroimaging

Antibodies

Antibodies used	biotin-conjugated anti-CD3 145-2C11, biotin-conjugated anti-CD19 6D5, biotin-conjugated anti-NK1.1 PK136, biotin-conjugated anti-Ly6G 1A8, biotin-conjugated anti-Ter119 TER-119, biotin-conjugated anti-F4/80 BM8, Alexa Fluor 700-conjugated anti–I-A/I-E M5/114.15.2, PerCP/Cy5.5-conjugated anti-Ly6C HK1.4, PE/Cy7-conjugated anti-CD11b M1/70, PE/Cy7-conjugated Annexin V, PE-conjugated anti-CD98 RL388, Brilliant Violet 711-conjugated anti-CD64 X54-5/71, APC-conjugated anti-F4/80 BM8, Pacific Blue-conjugated anti-CD29 HMβ1-1, Alexa Fluor 700-conjugated anti-CD3 17A2, Brilliant Violet 510-conjugated anti-CD4 GK1.5, PerCP or PerCP/Cy5.5-conjugated anti-CD8 53-6.7, APC/Fire 750-conjugated anti-CD11c N418, APC-conjugated anti-Ly6G 1A8, Brilliant Violet 785-conjugated anti-CD19 6D5, APC-conjugated anti-CD207 4C7, PE/Dazzle-conjugated anti-CD115 AFS98, APC-conjugated anti-CD135 A2F10, PE/Dazzle-conjugated anti-CD14 Sa14-2, PerCP/Cy5.5-conjugated CD81 Eat-2 (all BioLegend), APC-conjugated anti-CCR2 475301 (R&D Systems), eVolve 655-conjugated anti-CD45 30-F11 (eBioscience), Brilliant Blue 515-conjugated anti-CD117 2B8, Brilliant Violet 786-conjugated anti-CD72 K10.6 (all BD Bioscience), Alexa Fluor 488-conjugated anti-phospho-S6 ribosomal protein (Ser235/236) D57.2.2E, unconjugated anti-phospho-p70S6 Kinase (Thr389) 108D2, PE-conjugated anti-rabbit IgG (all Cell Signaling), unconjugated anti-Zeb2 CUK2 (provided by E.S.), DyLight 405-conjugated anti-rabbit IgG (Jackson ImmunoResearch).
Validation	Antibodies were used in consideration of the manufacture's recommendations and titrated before use. Specific validations have not been carried out.

Animals and other organisms

Policy information about studies involving animals; ARRIVE guidelines recommended for reporting animal research

Laboratory animals	Cx3cr1-GFP (B6.129P-Cx3cr1tm1Litt/J) and Cx3cr1CreER (B6.129P2(Cg)-Cx3cr1tm2.1(cre/ERT2)Litt/WganJ) were obtained from the Jackson Laboratory (Bar harbor, ME). CD98hcflox/flox were provided by Hideki Tsumura and Morihiro Ito. C57BL/6 , B6.Ly5.1 (B6.SJL-Ptprca Pepcb/BoyJ) and CCR2-/- (B6.129S4-Ccr2tm1Ifc/J) strains were bread at the animal facility of the University of

	Basel, Switzerland.
Wild animals	No wild animals were used
Field-collected samples	No Field-collected samples were used
Ethics oversight	Animal protocols were approved by the animal welfare committee Canton Basel Stadt (animal protocol #2854_27600)

Note that full information on the approval of the study protocol must also be provided in the manuscript.

Human research participants

Policy information about studies involving human research participants

Population characteristics	Patients diagnosed with Crohn's disease or ulcerative colitis were included into the study. Detailed patient characteristics are given in supplementary table 3 and in supplementary table 4.
Recruitment	Healthy and inflammatory bowel disease patients were recruited at the University Hospital Basel, Switzerland (Basel IBD Cohort). Biopsies were also obtained from the SwissIBD Cohort which is a national Swiss IBD cohort to which Swiss gastroenterologist include patients. The project has been approved by the Swiss IBD Cohort scientific commitee (SwissIBD cohort project 2016-12).
Ethics oversight	The Ethics Committee for Northwest and Central Switzerland (EKNZ) approved the protocol (EKBB 139/13 (PB 2016.02242))

Note that full information on the approval of the study protocol must also be provided in the manuscript.

Flow Cytometry

Plots

Confirm that:

☒ The axis labels state the marker and fluorochrome used (e.g. CD4-FITC).

☒ The axis scales are clearly visible. Include numbers along axes only for bottom left plot of group (a 'group' is an analysis of identical markers).

☒ All plots are contour plots with outliers or pseudocolor plots.

☒ A numerical value for number of cells or percentage (with statistics) is provided.

Methodology

Sample preparation

Isolation of bone marrow cells
After the preparation of femurs and tibias, connective tissues and muscles were removed and the bones were opened at the epiphysis. A syringe with a 25-gauge needle was placed into the ends of the opened femurs and tibias. Bone marrow cells were flushed out with RPMI 1640 medium (Sigma). The collected cells were passed through a 70 µm cell strainer to remove cell clumps and bone fragments.

Colonic lamina propria cell isolation
The isolated colon was opened longitudinally and washed with PBS to remove debris and mucus. The intestinal epithelium was removed by incubation in 5 mM EDTA in Ca2+/Mg2+-free PBS at 37°C under gentle shaking for 10 min for a total of three incubations. After every incubation cycle the tubes were vortexed for 30 s and the tissue pieces were transferred into fresh EDTA/PBS. The colon was washed in PBS to remove residual EDTA. The tissue was cut as small as possible and digested with 0.5 mg/ml Collagenase type VIII (Sigma-Aldrich) and 10 U/ml DNase (Roche) in RPMI 1640 for 20-25 min at 37°C in a water bath with continuous shaking (200 rpm). Every 5 min, the tubes were vortexed manually for 30 s. Supernatants were collected and passed through a 70 mm cell strainer, and cLP cells were pelleted by centrifugation. The cells were counted and processed for flow cytometry analysis.

Microglia
Mouse brain was prepared, chopped into small pieces, and passed through a 70 µm cell strainer. Then, the microglia was enriched using the CD11b (Microglia) MicroBeads Kit (Miltenyi Biotec) according to the manufacturer's protocol.

Cardiac macrophage
At least five minuted before the animals were sacrificed the mice, circulating leukocytes were labelled by intravenously injection of 1 µg anti-mouse CD45-Superbright in a final volume of 200 µl PBS per mouse using an insulin syringe. Theheart was prepared by open the chest under the sternum after cutting through the ribs. The right atrium was then opened followed by gently perfusion of the heart through the apex with 20 ml of cold 1X PBS (~7-9 ml/min). Then the right ventricle was perfused. Afterwards, the atria were dissected from the ventricles and discarded. The isolated ventricles were then transferred into 1.5 ml tubes containing 1 mL cold digestion medium (1X PBS with 100 µg/ml collagenase IV (Sigma)), in which the heart was cut into small pieces. TFor digestion, the samples were incubated at 37°C for 45 min with gentle shaking (~ 30 rpm) in digestion medium. Samples were mechanically homogenized by up and down motions through a 1 ml syringe capped with an 18 G needle. Afterwards, the samples were transferred into a 50 ml tube through a 70 µm cell strainer to remove the tissue stroma. Cells were pelleted by centrifugation. Supernatant was discarded and erythrocytes were eliminated by blood cell lysis buffer for ~3 min. Cells were washed with 10 ml FACS buffer.

Yolk sac cell isolation
The yolk sac (YS) was harvested from embryos at E8.5. Embryos were exsanguinated through decapitation in PBS containing 3% fetal calf serum (FCS, Gibco). To obtain single-cell suspension the YS was incubated in RPMI 1640 medium containing 1 mg/ml collagenase type VIII, 100 U/ml DNase I and 3% FCS at 37°C for 30 min. The digested YS was poured through a 70 µm cell strainer and erythrocytes were lysed (3–5 min at RT with Tris-Lysing buffer (144 mM NH4Cl, 17 mM Tris)). Cells were counted and processed for flow cytometry analysis.

Liver perfusion and liver cell isolation
The portal vein of an anesthetized (Isoflurane) animal was punctured with a 25-gauge needle. The liver was perfused with 10 ml liver perfusion medium (Gibco) followed by 5 ml liver digest medium (Gibco) after cutting the lower vena cava. Following removal of the gallbladder, the liver was placed into a petri dish and cut into small pieces. The tissue pieces were transferred into a 50 ml tube containing 5 ml liver digest medium and digested for 30 min at 37°C. Afterwards, the digested tissue was poured and mashed through a metal cell strainer to remove connective tissue and centrifuged for 5 min and 500 rpm at RT. The supernatant (solution A) and pellet (solution B) were separated into two tubes. The solution A was centrifuged for 5 min and 1400 rpm at RT. To solution B 40 ml PBS was added and centrifuged for 5 min and 500 rpm at RT. The supernatant from solution A was discarded and the supernatant form solution B was added to the pellet of solution A. After centrifugation for 5 min and 1400 rpm at RT, the supernatant was discarded and the pellet frothed up with 3 ml of PBS/2% FBS supplemented with 0.1% w/v sodium azide and 10 mM EDTA and 3.5 ml 70% Percoll (GE Healthcare) to obtain the 'Cell-Percoll-Suspension'. A Percoll gradient was prepared and centrifuged for 20 min and 2000 rpm without break. The fat layer on the top has been removed and the interphase which contains the lymphocytes and erythrocytes as well as the whole upper liquid phase to increase the cell yield were collected. After the erythrocytes were lysed (3–5 min at RT with Tris-Lysing buffer (144 mM NH4Cl, 17 mM Tris)), the cells were counte
d and processed for flow cytometry analysis.

Langerhans cell isolation
After cutting off the mouse ears, the ears are divided into dorsal and the ventral halves, from which the cartilage is removed with forceps. The ears were then placed dermal side down onto PBS containing 2.5 mg/ml dispase II (Sigma) and were incubated for 2 hours at 37°C. The dissociated epidermal sheets are placed in stop medium (2% FCS in PBS) and further transferred into a 50 ml tube with 20 ml RPMI 1640 medium containing 10% FCS and supplemented with 0.05mM 2-ME, 100 U/ml penicillin, and 100 mg/ml streptomycin. To release the Langerhans cells (LCs), the tube was gently shaking for 30 min at 37°C in a water bath. The remaining epidermal pieces and cell suspension were filtered through a 70 µm cell strainer and cells pelleted by centrifugation for 5min and 1400 rpm at 4°C. The cells were counted and processed for flow cytometry analysis.

Bone marrow-derived macrophages
Murine bone marrow cells were cultured in 6-well plates in RPMI 1640 medium containing 10% FCS and supplemented with 0.05 mM 2-ME, 100 U/ml penicillin and 100 µg/ml streptomycin. Macrophages were generated by adding 20 ng/ml M-CSF (BioLegend). After 7 days, macrophages were either stimulated with 100 ng/ml Lipopolysaccharide (LPS) from Escherichia coli O111:B4 (Sigma) and 10 ng/ml recombinant mouse IFN-γ (rmIFN-γ; BioLegend) or with 10 ng/ml recombinant mouse IL-4 (rm IL-4; BioLegend) and 10 ng/ml recombinant mouse IL-13 (rmIL-13; BioLegend) for 6 hours before cells were analyzed. For in vitro CD98 silencing tamoxifen dissolved in DMSO (Roth) was added into the culture during the macrophage generating and during LPS + IFNγ or IL-4 + IL-13 stimulation or D-phenylalanine (Sigma) was added 1 hour prior and during LPS + IFNγ or IL-4 + IL-13 stimulation.

Instrument

BD LSRFortessaTM X-20 flow cytometer (BD Biosciences)
BD FACSAria™ III (BD Biosciences)

Software

FACSDiva and FlowJo

Cell population abundance

Target cell populations were determined according the gating strategy discribed in the manuscript

Gating strategy

FSC-A vs. FSC-H was used to discriminate singlet cells. FSC-A vs. SSC-A was used to exclude debris. All cell populations were first gated on viability and lineage negative cells.
Bone marrow: CD115+, MDPs were identified as, CD117+, CD135+, Ly6C-, and CD11b- cells. cMoPs were defined as CD117+, CD135-, Ly6C+ and CD11b- cells, and monocytes characterized as CD117-, CD135-, and CD11b+ cells with Ly6Chigh, Ly6Cmid, and Ly6Clow expression.
Colonic lamina propria cells: CD11b+, CCR2+, Ly6C high/MHC II-, Ly6C mid/MHC II+, Ly6C low/MHC II+ or CD64+, Ly6C-/MHC II-, Ly6C-/MHC II+. Additional apoptosis staining of characterized cell populations: Annexin V
Fetus macrophages were characterized as CD45+, F4/80+
CD11c-/CD11c+ liver myeloid cells were characterized as CD45+, F4/80+, CD11b+, CD11c-/CD11c+, Ly6c-, MHC II+
Langerhans cells: CD45+, F4/80+, MHC II+, CD207+

☒ Tick this box to confirm that a figure exemplifying the gating strategy is provided in the Supplementary Information.
